# Supplementary material for: Assembling Near-Infrared Dye on the Surface of Near-Infrared Silica-Coated Copper Sulphide Plasmonic Nanoparticles
Source: Nanomaterials (Basel). 2023 Jan 27;13(3):510. doi: 10.3390/nano13030510 (PMC9919055; doi:10.3390/nano13030510)
Supplement: Supplementary file 1 [file nanomaterials-13-00510-s001.zip › nanomaterials-2157086-supplementary.pdf]

nanomaterials

MDPI

Supplementary Materials

Assembling Near-Infrared Dye on the Surface of Near-Infrared Silica-Coated Copper Sulphide Plasmonic Nanoparticles

Oleg Dimitriev [1,2,*], Yuri Slominskii [3], Mariangela Giancaspro [4,5], Federica Rizzi [4,5], Nicoletta Depalo [5], Elisabetta Fanizza [4,5] and Tsukasa Yoshida [2]

1 V. Lashkaryov Institute of Semiconductor Physics NAS of Ukraine, pr. Nauki 41, 03028 Kyiv, Ukraine
2 Graduate School of Organic Materials Science, Yamagata University, Jonan 4-3-16, Yonezawa 992-8510, Japan
3 Institute of Organic Chemistry NAS of Ukraine, 5 Murmanska Str., 02660 Kyiv, Ukraine
4 Chemistry Department, University of Bari, via Orabona 4, 70125 Bari, Italy
5 CNR-Institute for Chemical and Physical Process, SS Bari, via Orabona 4, 70125 Bari, Italy
* Correspondence: dimitr@isp.kiev.ua

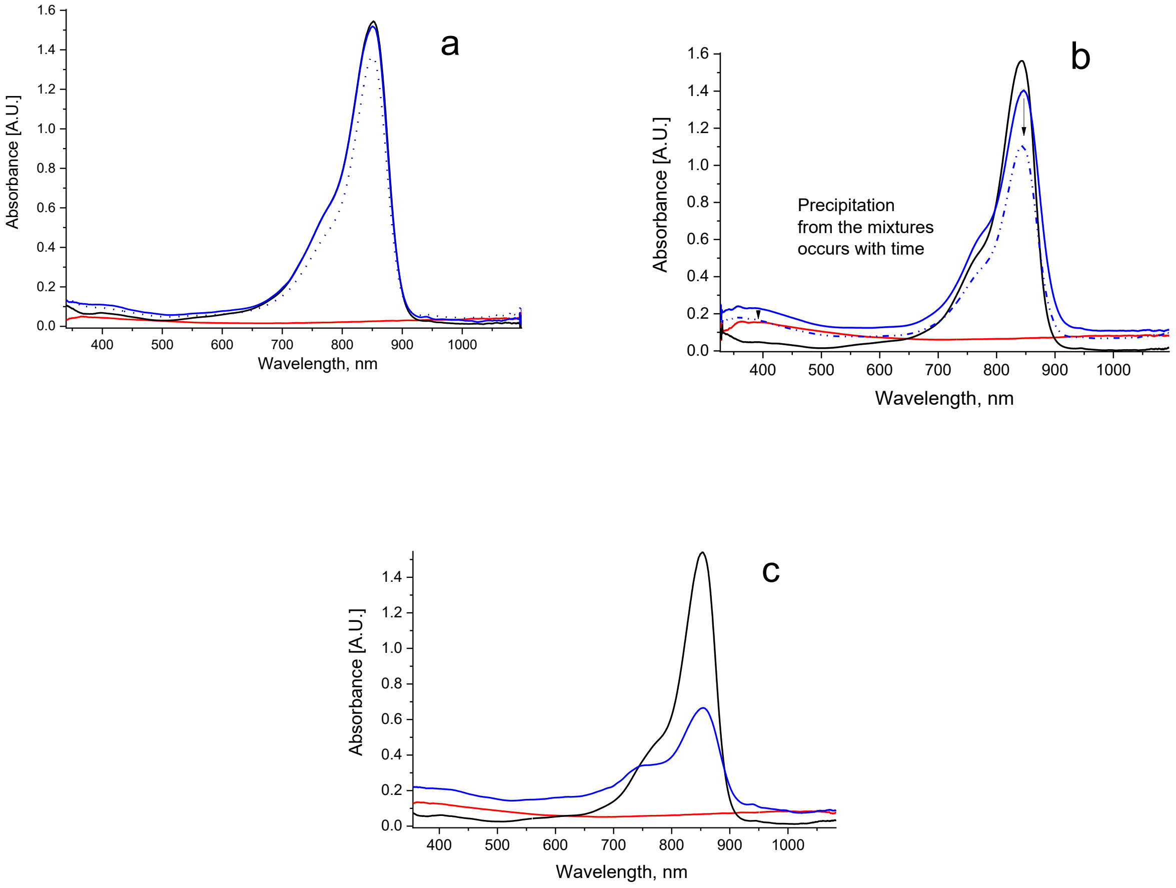

Figure S1. Absorption spectra of (**a**) DMSO, (**b**) IPA and (**c**) THF solution of dye (10^{-5} M, black curves), as-prepared dye:$Cu_{2-x}S$@MSS-NH_2 (0.14 g/L, blue solid), aged dye:$Cu_{2-x}S$@MSS-NH_2 (blue dotted) and $Cu_{2-x}S$@MSN-NH_2dispersion (red line), respectively.

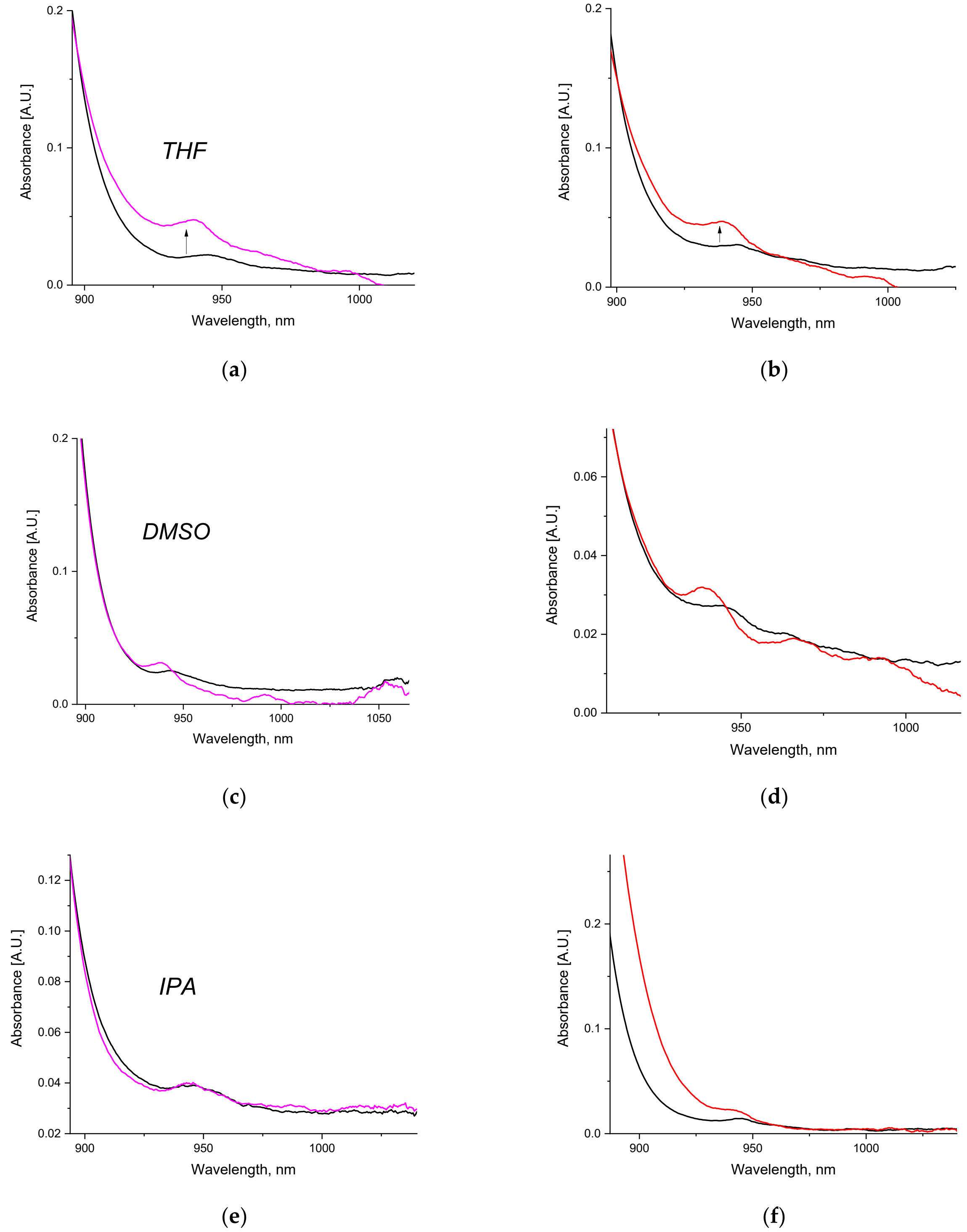

Figure S2. Spectra in the HBA region of the neat dye solution (black curves) and dye:$Cu_{2-x}S$@MSS (magenta curves) and dye:$Cu_{2-x}S$@MSS-NH_2 (red curves) hybrids corrected for the absorption of NPs for (**a**,**b**) THF, (**c**,**d**) DMSO, and (**e**,**f**) IPA solutions.

Table S1. Absorption wavelength and intensity of the monomer (mon) band and the blue-shifted shoulder (dim), and their relative absorption ratio for dye in ACN (10^{-5} M) and in the colloidal solution containing the $Cu_{2-x}S$@MSS NPs (0.15g/L).

	**Dye in Dispersant Solvent**					**Dye in Dispersant Solvent with $Cu_{2-x}S$@MSS**				
	λ_{mon} nm	λ_{dim} nm	Abs_{mon}	Abs_{dim}	Abs_{mon}/Abs_{dim}	λ_{mon} nm	λ_{dim} nm	Abs_{mon}	Abs_{dim}	Abs_{mon}/Abs_{dim}
ACN	833	759	1.5	0.6	2.5	833	759	1.4	0.6	2.3

Table S2. Specific heat capacity of the organic solvents used in this work.

	**THF**	**DMSO**	**ACN**	**IPA**
Specific heat capacity, J/g·K	1.77	1.96	2.24	2.68

Table S3. Polarity index and dielectric constant for the dispersant solvents used in this work.

	**Polarity Index**	**Dielectric Constant**
H_2O	10	80
DMSO	7.2	47
ACN	5.8	37
THF	4	17
IPA	3.9	17
